# Real-Time Loop-Mediated Isothermal Amplification (RealAmp) for the Species-Specific Identification of *Plasmodium vivax*

**DOI:** 10.1371/journal.pone.0054986

**Published:** 2013-01-22

**Authors:** Jaymin C. Patel, Jenna Oberstaller, Maniphet Xayavong, Jothikumar Narayanan, Jeremy D. DeBarry, Ganesh Srinivasamoorthy, Leopoldo Villegas, Ananias A. Escalante, Alexandre DaSilva, David S. Peterson, John W. Barnwell, Jessica C. Kissinger, Venkatachalam Udhayakumar, Naomi W. Lucchi

**Affiliations:** 1 Division of Parasitic Diseases and Malaria, Center for Global Health, Centers for Disease Control and Prevention, Atlanta, Georgia, United States of America; 2 Department of Genetics, University of Georgia, Athens, Georgia, United States of America; 3 Center for Tropical and Emerging Global Diseases, University of Georgia, Athens, Georgia, United States of America; 4 Waterborne Disease Prevention Branch, National Center for Emerging and Zoonotic Infectious Diseases, Centers for Disease Control and Prevention, Atlanta, Georgia, United States of America; 5 Asociación Civil Impacto Social, Tumeremo, Venezuela; 6 Arizona State University, Tempe Arizona, United States of America; 7 Institute of Bioinformatics, University of Georgia, Athens, Georgia, United States of America; Université Pierre et Marie Curie, France

## Abstract

*Plasmodium vivax* infections remain a major source of malaria-related morbidity and mortality. Early and accurate diagnosis is an integral component of effective malaria control programs. Conventional molecular diagnostic methods provide accurate results but are often resource-intensive, expensive, have a long turnaround time and are beyond the capacity of most malaria-endemic countries. Our laboratory has recently developed a new platform called RealAmp, which combines loop-mediated isothermal amplification (LAMP) with a portable tube scanner real-time isothermal instrument for the rapid detection of malaria parasites. Here we describe new primers for the detection of *P. vivax* using the RealAmp method. Three pairs of amplification primers required for this method were derived from a conserved DNA sequence unique to the *P. vivax* genome. The amplification was carried out at 64°C using SYBR Green or SYTO-9 intercalating dyes for 90 minutes with the tube scanner set to collect fluorescence signals at 1-minute intervals. Clinical samples of *P. vivax* and other human-infecting malaria parasite species were used to determine the sensitivity and specificity of the primers by comparing with an 18S ribosomal RNA-based nested PCR as the gold standard. The new set of primers consistently detected laboratory-maintained isolates of *P. vivax* from different parts of the world. The primers detected *P. vivax* in the clinical samples with 94.59% sensitivity (95% CI: 87.48–98.26%) and 100% specificity (95% CI: 90.40–100%) compared to the gold standard nested-PCR method. The new primers also proved to be more sensitive than the published species-specific primers specifically developed for the LAMP method in detecting *P. vivax*.

## Introduction

Malaria remains one of the top five causes of death in low income countries and is a major source of morbidity worldwide [1]. In 2010, the World Health Organization (WHO) estimated that 216 million clinical cases and 655,000 deaths were due to malaria infections [2]. Geographically, *Plasmodium vivax* is the most widely distributed human-infecting species, with up to 2.6 billion people at risk for infection [3]. Historically, *P. vivax* infections have been viewed as “benign” and self-limiting [4], [5]. However, recent evidence suggests that *P. vivax* infection causes significant morbidity including severe illness leading to mortality [5]–[7]. *P. vivax* can also co-occur with other *Plasmodium* species causing further complications for accurate diagnosis and treatment [8].

Early and accurate diagnosis of malaria is essential for case management and for malaria control programs [9]. Current diagnostic methods include microscopy, immunochromatographic rapid diagnostic tests (RDTs) that target parasite proteins, and polymerase chain reaction (PCR) -based molecular tests. Each method has advantages and limitations. Microscopy is considered the gold standard of malaria diagnosis in endemic countries. Correctly employed, it can identify different species and quantitate infection levels. While inexpensive, microscopy is time-consuming, labor-intensive, and it requires well-trained personnel along with appropriate infrastructure [10]. RDTs are easy to use, provide quick results, and are useful alternatives when there is no access to microscopic diagnosis [11]. One major limitation is that most RDTs cannot readily distinguish *P. vivax* from other species. Molecular diagnostic techniques based on DNA amplification such as PCR-based assays (standard, nested, and real-time) are highly sensitive and retain the ability to accurately differentiate between species [10]. However, since these methods require technical expertise, a well-equipped laboratory, and have long turnaround times, they are beyond the capacity of most malaria-endemic countries, making them difficult to implement in simple clinical laboratories or field settings.

The loop-mediated isothermal amplification (LAMP) method shows great potential for field use as it is simple and field-amenable [12]. DNA is amplified under isothermal conditions, removing the need for sophisticated and expensive thermal cyclers. LAMP employs autocycling strand displacement DNA synthesis using *Bacillus stearothermophilus* (*Bst*) DNA polymerase and a set of four primers that bind to unique sites on the target sequence making them highly specific [12]. The use of two loop primers shortens the time to product formation, thereby reducing overall reaction time to between 30 minutes and an hour [13]. LAMP has been utilized to detect malaria parasites mainly using the conventional 18S ribosomal RNA (18S rRNA) gene as the target sequence [14]–[18].

Recently, our laboratory developed a simple portable device that integrates the isothermal amplification technique (LAMP) with a fluorescent detection unit. This new platform, capable of real-time data collection, is referred to as RealAmp. It was used to successfully detect malaria parasites using published *Plasmodium* genus-specific primers [19]. In this study, we have successfully developed and tested novel *P. vivax*-specific LAMP primers on the RealAmp platform. These primers target dispersed genomic repeats in the *P. vivax* genome. Repeats were identified using recently developed diagnostic target mining methods [20].

## Methods

### Ethics Statement

Clinical samples were retrospectively used in this investigation that were obtained from a molecular surveillance study conducted in Venezuela. This study was originally approved by the institutional ethical review board of Instituto de Altos Estudios Dr. Arnoldo Gabaldon, Maracay, Aragua State, Venezuela. A written consent was obtained from the participants. Some of the clinical samples were obtained from a malaria reference laboratory in the Centers for Disease Control and Prevention (CDC).

### *Plasmodium* clinical samples and laboratory isolates

We used laboratory-adapted malaria parasites and clinical samples obtained from two different sources to test the novel primers. The *P. falciparum* (3D7) isolate, and other human malaria parasites used in this study such as *P. vivax, P. malariae, P. ovale* and *P. knowlesi* were available in the CDC malaria branch collection. Thirteen geographically diverse *P. vivax* strains (Miami, El Salvador, Honduras, Mauritania, Indonesia, India, South Vietnam, North Korea, Thailand, New Guinea, Panama, Chesson, and Pakchong) were included. Due to genetic similarity between *P. vivax* and some simian malaria parasites, we also tested eight simian malaria parasites, *P. simiovale, P. inui, P. coatneyi, P. hylobati, P. fragile, P. gonderi, P. fieldi*, and *P. cynomolgi*. To determine the limits of detection, we used two fold-serial dilutions of two *P. vivax* strains (Brazil and India) starting from 2000 parasites/µl to 0.98 parasites/µl. Sixty eight *P. vivax* clinical samples were obtained from a previous surveillance study conducted in Venezuela. In addition, DNA from 52 clinical samples from our reference diagnostic laboratory, previously diagnosed using a nested-PCR method (10 malaria-negative samples, 14 *P. falciparum*, 9 *P. vivax,* 1 *P. malariae*, 12 *P. ovale, 2 P. falciparum/P. malariae, 1 P.vivax/P.ovale, 2 P. falciparum/P. ovale* mixed infections and 1 *P. knowlesi*), were included to test for specificity and sensitivity.

### DNA extraction

DNA from blood samples was extracted using the QIAamp DNA minikit (Qiagen, Valencia CA) according to the manufacturer's recommendations. All extracted samples were aliquoted and stored at −20°C.

### Nested PCR

Nested PCR designed to amplify 18S ribosomal RNA gene sequences was performed as previously described [19], [21]. Briefly, 20 µl amplification reactions containing 1X buffer, 2.5 mM MgCl_2_, 200 µM dNTPs, 200 nM primers, and 1.25 units of Taq Polymerase (New England Biolabs, Ipswich, MA) were performed on all samples. Nested-PCR products were analyzed via gel electrophoresis (2% gel) in order to visualize and size the bands.

### Data harvesting

Demas *et al*. applied highly specific criteria to screen and identify unique target sequences for *P. vivax* and *P. falciparum* through genome sequence data mining [20]. We used two previously identified *P. vivax* sequences (Pvr47 and Pvr64) to design novel *P. vivax-*specific LAMP primers. Several potential *P. vivax* primers were designed using Eiken's PrimerExplorer software v3 (http://loopamp.eiken.co.jp/e/lamp/primer.html). Five primer sets were tested on the RealAmp platform at temperatures ranging from 60°C to 64°C. Primer sets with the shortest amplification time that correctly amplified *P. vivax* with no cross-reactivity with other human-infecting or primate *Plasmodium* species were selected for further analysis.

### RealAmp Method

The RealAmp method used for this study has been described in detail [19]. Briefly, the LAMP assays were performed in a 12.5 µL total volume containing a 2X in-house buffer (40 mM Tris-HCl pH 8.8, 20 mM KCl, 16 mM MgSO_4_, 20 mM (NH_4_)SO_4_, 0.2% Tween-20, 0.8 M Betaine, 2.8 mM of dNTPs each), 0.25 µL of a 1∶100 dilution SYBR green, 8 units of *Bst* DNA polymerase (New England Biolabs, Ipswich, MA), and 2 µl of template DNA. Amplifications were carried out at 64°C for 90 minutes using the ESE-Quant Tube scanner (Qiagen, USA) collecting fluorescence signals at 1-minute intervals. All samples were run at least twice using the r64 primer set as well as published *P. vivax* LAMP primers [15]. If the two runs were discordant, the sample was run a third time and a criterion of two concordant results out of three runs was used to make the final call. We used a cutoff of 60 minutes and any sample that amplified after this cutoff point was considered to be negative. Since SYBR Green has known potential inhibitory effects on DNA amplification [22], [23], all samples were also tested using 1∶400 dilution of SYTO-9 fluorescent dye.

### Statistical Analysis

A total of 120 and 118 samples were included in the sensitivity and specificity analyses of the novel Pvr64 and published *P. vivax* primers [15], respectively. Nested PCR as described by Singh *et al*. provided a gold standard [21]. Specificity and sensitivity were calculated as follows:

*Sensitivity  =  true positives/(true positives + false negatives) X 100.*

*Specificity  =  true negatives/(true negatives + false positives) X 100.*

95% confidence intervals (95% CI) were also calculated.

## Results

### Novel candidates for primer design

The Pvr47 sequence is described in Demas *et al* [20]. Pvr64 is a highly conserved repeat family (>98% identity) present in six copies in the *P. vivax* genome. Five copies are on small contigs that were not assembled into chromosome scaffolds because of their repetitive nature [24]. The remaining copy is on chromosome 9, ∼500 kb from the end of the assembly. Three of the copies are located inside the annotated proteins PVX_092555, PVX_246295, and PVX_238290. PVX_092555 is *Plasmodium*-specific and has orthologs in the human-infecting species *P. falciparum* and *P. knowlesi*, as well as the rodent-infecting species *P. berghei, P. chabaudi*, and *P. yoelli*. PlasmoDB does not contain orthologs for PVX_246295 or PVX_238290 [25], but both appear to be truncated paralogs of PVX_092555 based on their nearly identical sequences.

The Pvr64 family is contained within the WD40 domain of *P. vivax* proteins and most of its *Plasmodium* orthologs. WD40 domains are found in many proteins implicated in a variety of functions across eukaryotes, including signal transduction, transcriptional regulation, and cell-cycle control. Annotations for most PVX_092555 orthologs contain little functional information, other than to note that they are conserved across the genus *Plasmodium*. One *P. yoelli* ortholog is thought to be a transcription factor-associated protein, while another *P. yoelli* ortholog (which has very low sequence similarity to PVX_092555 and does not contain a WD40 domain) is annotated as an erythrocyte membrane protein.

The Pvr64 family has a high degree of identity with its *Plasmodium* orthologs (nucleotide identity ranges from 68% in *P. falciparum* to 86% in *P. knowlesi*). Because of this high similarity, the Pvr64 segment used to design the novel primers was aligned with the *P. knowlesi* and *P. falciparum* sequences and the locations of the six LAMP primers are indicated. Several nucleotide differences were observed within the region used for the six primers (Figure S1).

### *P. vivax* primers

Five primer sets were designed from the two selected target candidates (Pvr64 and Pvr47). Each primer set was first tested with all known human infecting *Plasmodium* species. If it was able to correctly amplify *P. vivax* and no other species, then we evaluated it on geographically diverse strains. Based on these criteria, only one primer set based on the Pvr64 target (r64) was selected for further validation (Table 1). The other four primers did not give consistent amplification of the *P. vivax* DNA and resulted in either very late amplification or negative results, implying that these were not good enough.

**Table 1:** *P. vivax* r64 RealAmp primer set sequence (5′→3′).

F3	TCT GTT GGT GGA GTA GAT CC
B3	CCT ACG TTT TGG TGA ATC G
FIP	ATA TGG TCT CTC GAC ACG GC – CAA ATT GCC ATC ATC TTC AC
BIP	TGT GCC CAC CCA CAT ACT TA – GGG AAA TGT TAA TGG GGA TGT
LF	AGG CTA CTT CTT TTG CTC C
LB	ACT TAC AGT GCT GTA GAG A

**Figure 1:**
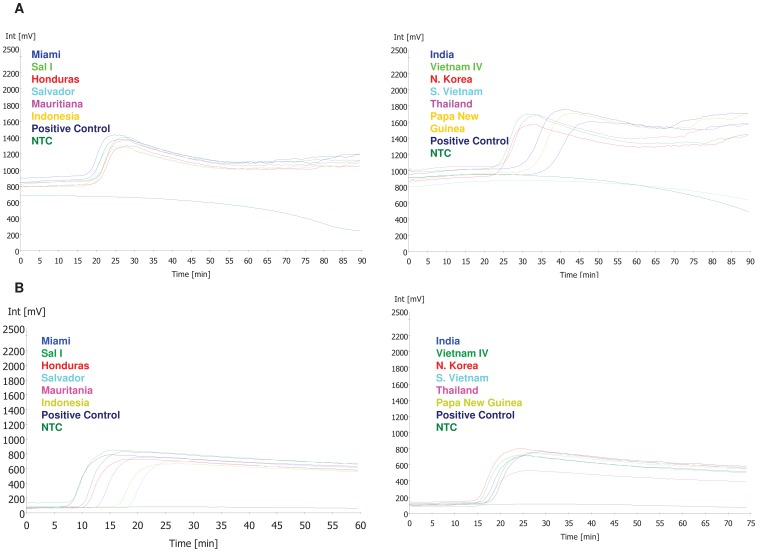
Amplification plots for *P. vivax* strains from different geographic locations using the r64 primer set with SYBR green (A) and SYTO-9 (B). Amplification plots from *P. vivax* strains from Pakchong, Chesson and Panama are not shown. Amplification of the template DNA measured in millivolts (mV) on y-axis over time on x-axis. Any amplification observed after 60 minutes of test time was considered negative. A known *P. vivax* DNA sample was used as the positive control. NTC; No Template Control.

We found that HPLC purification of the primers provided better amplification plots with reduced background noise (data not shown). The time to amplification for all samples tested ranged from 18 to 49 minutes from the start of the reaction (Figure 1).

### Specificity of *P. vivax* specific LAMP primers

We tested the novel primer sets on all known human malaria parasites (*P. vivax, P. falciparum, P. malariae, P. ovale*, and *P. knowlesi*). Only *P. vivax* was amplified (Table 2). In addition, no cross reactivity was observed with any of the tested simian malaria parasite species or with the malaria-negative human DNA control (Table 2).

**Table 2:** Specificity results of the r64 primer set when tested against human-infecting and simian-infecting malaria parasites.

Human and simian malaria parasite species	RealAmp Result
***P. falciparum***	−
***P. vivax***	+
***P. ovale***	−
***P. malariae***	−
***P. knowlesi***	−
***P. simiovale***	−
***P. inui***	−
***P. coatneyi***	−
***P. hylobati***	−
***P. fragile***	−
***P. gonderi***	−
***P. fieldi***	−
***P. cynomolgi***	−
***Malaria-negative human DNA***	−

All species were tested at least twice for reproducibility.

In order to validate the specificity of primer set r64, *P. vivax* strains from different geographic regions (Miami, El Salvador, Honduras, Mauritania, Indonesia, India, South Vietnam, North Korea, Thailand, New Guinea, Panama, Chesson, and Pakchong) were tested. We were able to amplify all of the different strains using our standard protocol (Figure 1 and data not shown).

### Limits of detection, sensitivity and specificity of LAMP primers in clinical samples

Using serial dilutions of two *P. vivax* strains from Brazil and India, the limit of detection of r64 primer set was determined to be 125 parasites/µl and the limit of detection of previously published *P. vivax* LAMP primers (Han *et al*.) was determined to be between 250–500 parasites/µl. Clinical samples were used to determine primer sensitivity and specificity. The r64 primer set accurately detected 70/74 *P. vivax*-positive samples. No amplification was observed with 46 *P. vivax*-negative samples. Compared to a nested PCR reference, r64 sensitivity and specificity are 94.59% (95% CI: 87.48–98.26%) and 100% (95% CI: 90.40–100%) respectively (Table 3). This is a drastic improvement over the sensitivity of previously published *P. vivax* LAMP primers (36.11% (95% CI: 25.37–48.35%) with a specificity of 100% (95% CI: 90.40–100%)) in our hands.

**Table 3:** Sensitivity and specificity among clinical sample of r64 primer set and published *P. vivax* LAMP primers.

Nested PCR (n)	Pvr64	
	Positive	Negative
**Positive (74)**	70	4
**Negative (46)**	0	46
**Sensitivity**	94.59% (95% CI: 87.48–98.26%)	
**Specificity**	100% (95% CI: 90.40–100%)	

Nested PCR (n)	Han *et al*. *P. vivax* LAMP primers [15]	
	Positive	Negative
**Positive (72)**	26	46
**Negative (46)**	0	46
**Sensitivity**	36.11% (95% CI: 25.37–48.35%)	
**Specificity**	100% (95% CI: 90.40–100%)	

Nested PCR using published 18S rRNA primers was used as the gold standard. All samples were tested at least twice for reproducibility.

### SYBR Green versus SYTO-9

The specificity and sensitivity of the *P. vivax* r64 assay with SYTO-9 fluorescent dye was found to be the same as that of the assay with SYBR Green. In addition, the SYTO-9 amplification plots had lower background noise and in some cases, a better distinction from baseline fluorescence than the SYBR Green assays (Figure 1).

## Discussion

Though the global distribution and disease burden of *P. vivax* is considerable, current molecular diagnostic methods that accurately detect *P. vivax* are limited. The RealAmp platform is a potential tool for using molecular diagnostic techniques in the field. The assay is simple, and provides rapid results through real-time data collection. This combination is ideal for developing countries that may not have the capacity and infrastructure to maintain PCR-based assays at every health care facility. The objective of this study was to design and test the utility of a novel set of LAMP *P. vivax*-specific primers using different target DNA sequences. The previously described genome sequence data-mining methods [20], adapted in this study, have proved to be useful in designing novel specific primers for *P. vivax* detection. Recent studies have developed new *P. falciparum* diagnostic LAMP primers based on mitochondrial DNA [26] and the apical membrane antigen-1 (AMA-1) gene sequence [27] was used to amplify *P. knowlesi* DNA. These new targets will help optimize the RealAmp assay for accurate detection of all malaria parasite species.

We have noted previously that *Plasmodium* subtelomeric regions harbor repetitive regions that are well suited to species-specific diagnostic targets [20], [28]. As the majority of Pvr64 copies are not assembled onto chromosomes, and the short assemblies containing these copies do not contain telltale signs of subtelomeric regions such as *vir* genes, we cannot be certain whether or not Pvr64 is a subtelomeric repeat. However, one chromosomal copy does occur ∼ 500 kb interior to the end of the assembly, arguably outside of the nebulously defined “subtelomeric” region. Though Pvr64 has some overlapping regions in other *Plasmodium* parasites such as *P. falciparum* and *P. knowlesi*, the primers developed in this study did not show any cross reactivity with other examined malaria parasites. While Pvr64 is similar to the 18S rRNA target in that it is relatively low-copy and not species-specific, it offers a superior, more sensitive RealAmp target than the 18S rRNA gene family, perhaps due to its comparatively high degree of conservation within the *P. vivax* genome.

The r64 primer set has shown reasonable sensitivity and specificity in detecting *P. vivax* among the clinical isolates used, accurately detecting 70 out of the 74 positive samples. It is possible that the four *P. vivax* samples that this primer set missed had low parasite density. Unfortunately, we were not able to determine this since we did not have any parasitemia data for the samples used in this study. Since *P. vivax* is genetically similar to several simian malaria parasites, we also tested the r64 primer set on eight simian parasites and observed no cross-reactivity. These primers were also able to amplify 13 *P. vivax* strains from different geographic locations. It is therefore likely that this assay will be able to detect *P. vivax* strains in malaria-endemic regions around the world.

Attempts to use published *P. vivax* LAMP primers by Han *et al*. in the RealAmp platform failed to yield consistent results, and the sensitivity was not as high as previously reported [15]. Possible explanations for this observation is the fact that different nested-PCR methods were used as the gold standard in calculating the sensitivity and specificity and that different detection methods were used in these studies. We also tested another published set of *P. vivax* LAMP primers by Tao *et al* [29]. However, these primers cross reacted with the other human-infecting *Plasmodium* species (*P. falciparum, P. ovale* and *P. malariae*), hence were not considered for further evaluation.

In some studies, SYBR Green was shown to have inhibitory effects on DNA amplification when used in high concentrations [22], [23]. We did not observe any inhibitory effect with SYBR Green in our assays. However, we obtained better amplification plots with SYTO-9 with lower background noise and in some cases, a better distinction from baseline fluorescence. This suggests that SYTO-9 is a better dye than SYBR Green for RealAmp assays and is possibly due to perfect spectral match with the detector. This is an important component of the assay as it will reduce misclassification of cases and non-cases by the users in the field.

One of the limitations of this primer set is its low sensitivity. Our data suggests that the limit of detection of the r64 primer set is around 125 parasites/µL when a quantified standard was used. This level of sensitivity might be sufficient for case management of malaria but not for the detection of sub-clinical malaria cases which is important during the pre-elimination phase of malaria. Efforts are underway to improve these limits of detection to enable detection of lower parasitemia. However, compared to nested PCR as a gold standard, this method performed reasonably well with approximately 95% sensitivity in detecting the clinical samples in a single assay.

As previously described, the RealAmp assay [19] has several advantages that make it a good choice for field-based molecular diagnosis of malaria. However, some hurdles need to be overcome for the RealAmp assay, and any molecular assay, to be truly field-usable. For example, a major caveat of molecular assays is the sample preparation phase; many assays require that the template DNA is purified and isolated. While the use of boiled whole blood was shown to give decent results with the RealAmp assay [19], additional studies are required to further simplify this step. In addition, the cold chain requirement for reagents used by many molecular assays needs to be addressed. Studies are underway to investigate if the RealAmp reagents can be combined into a “kit” which would further simplify the protocol, reduce the number of steps for the end user, and minimize chances of user-introduced errors in performing this assay. Lyophilized reagents that can be reconstituted at a later time would reduce preparation time and address the issue of reagent storage without a cold chain supply.

In conclusion, this study demonstrates that the *P. vivax* LAMP primers developed here have the ability to accurately detect *P. vivax* infections on the RealAmp platform. Furthermore, the previously described genome sequence data-mining methods have proved to be useful in designing primers not just for conventional PCR [20] but also for LAMP assays. This technique offers great potential in identifying novel primer sets for detection of other *Plasmodium* species.

## Supporting Information

Figure S1 **Comparison of the three sequences.** The Pvr64 segment used to design the novel *P. vivax* primers was aligned with the *P. knowlesi* and *P. falciparum* sequences. The locations of the six LAMP primers are underlined (italicized sequences indicate location of the complementary sequences). Nucleotide differences found in the *P. knowlesi* (highlighted in green) and *P. falciparum* (highlighted in yellow) are shown.
(DOCX)
