# Supplementary material for: Real-Time Loop-Mediated Isothermal Amplification (RealAmp) for the Species-Specific Identification of Plasmodium vivax
Source: PLoS One. 2013 Jan 22;8(1):e54986. doi: 10.1371/journal.pone.0054986 (PMC3551762; doi:10.1371/journal.pone.0054986)
Supplement: Figure S1 — Comparison of the three sequences. The Pvr64 segment used to design the novel P. vivax primers was aligned with the P. knowlesi and P. falciparum sequences. The locations of the six LAMP primers are underlined (italicized sequences indicate location of the complementary sequences). Nucleotide differences found in the P. knowlesi (highlighted in green) and P. falciparum (highlighted in yellow) are shown. (DOCX) [file pone.0054986.s001.docx]

[F3] [F2] [LF]

**Pv** GGGGTATGTTCTGTTGGTGGAGTAGATCCTCAAATTGCCATCATCTTCA-CTGGAGCAAA

**Pk** GGGGTATGTTCTATTGGTAGAGTAGATCCTTAAATTACCATCATCTTCA-CTGGAACAAA

**Pf** TGGATAAGTTTTATTAGTAGAATATATTCTTAAATTTCCATCATCTTCCGCAGTTGC-GA

[F1c] [B1c]

**Pv** AGAAGTAGCCTAAGAAGGCCGTGTCGAGAGACCATATAGGTGTGTGCCCACCCACATACT

**Pk** AAAAGTAGCCTAAAAAGGCCGTGTCCAGAGACCATATAGGTGTGTGTCCACCTACATACT

**Pf** AGAAGTATCCTAAAAAGGCTGTGTCTATAGACCATATTGGTGTATGTCCACCTACATATT

[LB] [B2] **Pv** TGACATAATTTTTATT*ACTTACAGTGCTGTAGAGA*TAT*ACATCCCCATTAACATTTCCCG*

**Pk** TCACATAATTTTTGTTACTTATTGTGCTGTATAAATATACATCCCCATTAACATTTCCAG

**Pf** TTACATAATTTTTATTACTAATTGTGCTGTATAAATATATATCACCATTTAAATTACCAG

[B3c] **Pv** ATAGTAATATTTTATT*CGATTCACCAAAACGTAGG*CTCGTAACGTTATGAATATTTCCAT

**Pk** AGAGTAATATTTTATTCGATTCACCAAAACGTAAACTCGTAACATTAAAAACATTCCCAT

**Pf** ATAATAAAATTTTATCTGTTTCACCAAAGGACAAACTAGACACATTAAATGTATTTCCAT
